# Supplementary material for: Targeted antiviral treatment using non-ionizing radiation therapy for SARS-CoV-2 and viral pandemics preparedness: Technique, methods and practical notes for clinical application
Source: PLoS One. 2021 May 14;16(5):e0251780. doi: 10.1371/journal.pone.0251780 (PMC8121356; doi:10.1371/journal.pone.0251780)
Supplement: S1 File — (DOCX) [file pone.0251780.s001.docx]

**S1 File.**

**Derivations pertaining to dipolar mode spherical oscillations**

Based on Lamb’s theory [18], the frequency of the dipolar mode SPH (*l*=1, *n*= 0) for any spherical particle (Fig 1) can be estimated using the eigen value equation:

$$4\frac{j_{2}(\xi)}{j_{1}(\xi)}-\eta^{2}+2\frac{j_{2}(\eta)}{j_{1}(\eta)}\eta=0$$

where $\xi=\frac{2\pi\nu D}{V_{L}}$, $\eta=\frac{2\pi\nu D}{V_{T}}$ and $j_{l}(\eta)$ is the spherical Bessel function of the first kind. D denotes diameter of the particle and V*_L_* and V*_T_* are the sound velocities of longitudinal and transverse waves, respectively.

For any variable z and any integer m > 0; as z → 0, $j_{m}(z)$ ∼ $\frac{1}{2^{m}m!}z^{m}$

Therefore, as ($\frac{D}{V_{L}}$) → 0; $\xi$ → 0, $\eta$ → 0.

The order of the higher order terms being lesser than the first term, their significance in the computation is minimal and hence a first order approximation is applicable without much change for the final solution.

Thus, $j_{2}(\xi)$ ∼ $\frac{1}{2^{2}2!}\xi^{2}$, $j_{2}(\eta)$ ∼ $\frac{1}{2^{2}2!}\eta^{2}$, $j_{1}(\xi)$ ∼ $\frac{1}{2^{1}1!}\xi^{1}$ and $j_{2}(\eta)$ ∼ $\frac{1}{2^{1}1!}\eta^{1}$.

This gives $\frac{j_{2}(\xi)}{j_{1}(\xi)}=\frac{\frac{\xi^{2}}{8}}{\frac{\xi}{2}}$ = $\frac{\xi}{4}$ and $\frac{j_{2}(\eta)}{j_{1}(\eta)}=\frac{\frac{\eta^{2}}{8}}{\frac{\eta}{2}}$ = $\frac{\eta}{4}$

The eigen value equation $4\frac{j_{2}(\xi)}{j_{1}(\xi)}-\eta^{2}+2\frac{j_{2}(\eta)}{j_{1}(\eta)}\eta=0$ can hence be expressed as $4\frac{\xi}{4}-\eta^{2}+2\frac{\eta}{4}\eta=0$

Thus, $\eta^{2} =2\xi$

Also, $\eta=2\xi$ (since $\xi=\frac{2\pi\nu D}{V_{L}}$, $\eta=\frac{2\pi\nu D}{V_{T}}$ and $\frac{V_{L}}{V_{T}}=2$)

Therefore, ${(2\xi)}^{2} =2\xi$

gives $2\xi=1$

or $\frac{4\pi\nu D}{V_{L}}=1$

which gives $\nu=\frac{V_{L}}{4\pi D}$

As angular frequency $\omega=2\pi\nu$, resonant frequency $f$ is derived to be

$$f=\frac{V_{L}}{2D}$$

- (**Equation 1**)

**Calculations pertaining to virus charge status**

Using $Q$ = 1.95, *q* = 1.16 × 10^7^e, ω*_0_* = 2π × 8.22 GHz, $m^{*}$= 14.5 MDa, *σ_abs_* = 2.5 × 10^-3^ m^2^ and $\varepsilon_{r}$ = 67.13 (in PBS) [13] for influenza A subtype H3N2; and $Q$ = 2, ω*_0_* = 2π × (15 to 17) GHz, $m^{*}$= 55.63 MDa, and $\varepsilon_{r}$ = 20 (in lungs) [27] for SARS-CoV-2, equation 2

gives $\frac{1.95 \times q_{1}^{2}}{8.22 \times14.5 \times{\sigma_{abs}}_{1}\sqrt{67.13}}=\frac{2 \times q_{2}^{2}}{\left( 15 to 17 \right) \times55.63 \times{\sigma_{abs}}_{2}\sqrt{20}}$

gives $\frac{1.95 \times q_{1}^{2}}{976.56 \times{\sigma_{abs}}_{1}}=\frac{2 \times q_{2}^{2}}{\left( 3731.77 to 4229.34 \right) \times{\sigma_{abs}}_{2}}$

gives $0.001997\frac{q_{1}^{2}}{{\sigma_{abs}}_{1}}=(0.000536 to 0.000473)\frac{q_{2}^{2}}{{\sigma_{abs}}_{2}}$

gives $(3.72 to 4.22)\frac{q_{1}^{2}}{{\sigma_{abs}}_{1}}=\frac{q_{2}^{2}}{{\sigma_{abs}}_{2}}$

It was shown that Influenza A subtype H3N2 at 7.5x10^14^m^−3^ particle density (N) within a 1.25 mm long sensing zone (*L*) presented an experimental absorption cross section *σ_abs_* of 2.5 × 10^-3^ m^2^ [13]. Considering $\sigma_{abs}=\frac{-ln(1-\alpha)}{N x L}$ [13]; and assuming that $N_{1}$ particles with $\alpha_{1}$ absorbance gives ${\sigma_{abs}}_{1}= {\sigma_{abs}}_{2}$ for $N_{2}$ particles with $\alpha_{2}$ absorbance over the same sensing/exposure zone of L units length we get $\left( 3.72 to 4.22 \right) q_{1}^{2}=q_{2}^{2}$ , where $N_{2}= \frac{-ln(1- \alpha_{2})}{-ln(1- \alpha_{1})}$ $N_{1}$.

Therefore, $q_{2}=\sqrt{\left( 3.72 to 4.22 \right)} q_{1}$

giving $q_{2}=(1.93 to 2.05) q_{1}$

giving $q_{2}\sim2 q_{1}$

giving $q_{2}$ ∼2.32 x 10^7^*e*.

**Calculations pertaining to threshold electric field intensities to fracture viral particles**

Using Equation 3 with $P_{Stress}^{T}$ = 0.141 MPa and *r* = 50 nm for influenza A subtype H3N2 [13], and $P_{Stress}^{T}$ = 0.141 MPa (ref. results) and *r* = (35 to 40) nm (ref. results) for SARS-CoV-2 along with the values for $m^{*}$, ω*_0_*, *q* and *Q* representing the same parameters as described above

$$\frac{{P_{Stress}^{T}}_{1} {r^{2}}_{1}\sqrt{{m^{*}}_{1}\left( {\omega_{0}^{2}}_{1}-{\omega^{2}}_{1} \right)+{(\frac{{\omega_{0}}_{1}{m^{*}}_{1}}{Q_{1}})}^{2}{{(\omega}^{2}}_{1})}}{{E_{T}}_{1}q_{1}{m^{*}}_{1}{\omega_{0}^{2}}_{1}}=\frac{{P_{Stress}^{T}}_{2} {r^{2}}_{2}\sqrt{{m^{*}}_{2}\left( {\omega_{0}^{2}}_{2}-{\omega^{2}}_{2} \right)+{(\frac{{\omega_{0}}_{2}{m^{*}}_{2}}{Q_{2}})}^{2}{{(\omega}^{2}}_{2})}}{{E_{T}}_{2}q_{2}{m^{*}}_{2}{\omega_{0}^{2}}_{2}}$$

gives ${E_{T}}_{2}={E_{T}}_{1}\frac{{P_{Stress}^{T}}_{2} q_{1}{m^{*}}_{1}{\omega_{0}^{2}}_{1}{r^{2}}_{2}\sqrt{{m^{*}}_{2}\left( {\omega_{0}^{2}}_{2}-{\omega^{2}}_{2} \right)+\left( \frac{{\omega_{0}}_{2}{m^{*}}_{2}}{Q_{2}} \right)^{2}{{(\omega}^{2}}_{2})}}{{{P_{Stress}^{T}}_{1}q}_{2}{m^{*}}_{2}{\omega_{0}^{2}}_{2}{r^{2}}_{1}\sqrt{{m^{*}}_{1}\left( {\omega_{0}^{2}}_{1}-{\omega^{2}}_{1} \right)+\left( \frac{{\omega_{0}}_{1}{m^{*}}_{1}}{Q_{1}} \right)^{2}{{(\omega}^{2}}_{1})}}$

gives ${E_{T}}_{2}={E_{T}}_{1}\frac{1.16 \times14.5 \times{(2\pi)}^{2}\times{(8.22)}^{2} \times\left( 35 to 40 \right)^{2}\sqrt{\left( \frac{2\pi\times\left( 15 to 17 \right) \times55.63}{2} \right)^{2}{\{2\pi\times\left( 15 to 17 \right)\}}^{2}}}{2.32 \times55.63 \times{{(2\pi)}^{2}\times(15 to 17)}^{2} \times{(50)}^{2}\sqrt{\left( \frac{2\pi\times8.22 \times14.5}{1.95} \right)^{2}{(2\pi\times8.22 )}^{2}}}$(when ω*_0_* = ω and since ${P_{Stress}^{T}}_{1}$ = ${P_{Stress}^{T}}_{2}$ in this case)

gives ${E_{T}}_{2}={E_{T}}_{1}\frac{1136.5 \times\left( 35 to 40 \right)^{2}\sqrt{\left( 27.81 \right)^{2} \times{\{2\pi\times\left( 15 to 17 \right)\}}^{4}}}{322654 \times{(15 to 17)}^{2}\sqrt{\left( 7.43 \right)^{2}{(2\pi\times8.22 )}^{4}}}$

gives ${E_{T}}_{2}={E_{T}}_{1}\frac{1136.5 \times\left( 35 to 40 \right)^{2} \times27.81 \times\sqrt{{\{2\pi\times\left( 15 to 17 \right)\}}^{4}}}{322654 \times{(15 to 17)}^{2} \times7.43 \times\sqrt{{(2\pi\times8.22 )}^{4}}}$

gives ${E_{T}}_{2}={E_{T}}_{1}\frac{31606.06 \times\left( 35 to 40 \right)^{2}\sqrt{\left( 15 to 17 \right)^{4}}}{2397319.22 \times{(15 to 17)}^{2} \sqrt{{(8.22 )}^{4}}}$

gives ${E_{T}}_{2}={E_{T}}_{1}\frac{31606.06 \times\left( 35 to 40 \right)^{2}\left( 15 to 17 \right)^{2}}{2397319.22 \times{(15 to 17)}^{2} {(8.22 )}^{2}}$

Therefore, at *r* = 35 nm with ω*_0_* = 2π × 17 GHz ${E_{T}}_{2}={E_{T}}_{1}\frac{31606.06 \times\left( 35 \right)^{2}\left( 17 \right)^{2}}{2397319.22 \times{(17)}^{2} {(8.22 )}^{2}}$

giving ${E_{T}}_{2}=0.24 {E_{T}}_{1}$

And at *r* = 40 nm with ω*_0_* = 2π × 15 GHz ${E_{T}}_{2}={E_{T}}_{1}\frac{31606.06 \times\left( 40 \right)^{2}\left( 15 \right)^{2}}{2397319.22 \times{(15)}^{2} {(8.22 )}^{2}}$

giving ${E_{T}}_{2}=0.31 {E_{T}}_{1}$

Hence ${E_{T}}_{2}\sim$ 0.275 ${E_{T}}_{1}$

or ${E_{T}}_{2}\sim$23.9 V/m
